# Supplementary material for: Exosomes combined with extra virgin olive oil reduces lipogenesis, oxidative stress, and inflammation in non-alcoholic fatty liver disease model
Source: PLoS One. 2025 Oct 27;20(10):e0333698. doi: 10.1371/journal.pone.0333698 (PMC12558531; doi:10.1371/journal.pone.0333698)
Supplement: Attachment — Submitted filename: Responses to reviewers.pdf [file pone.0333698.s002.pdf]

Dear Reviewer, 1,

Thank you for the review of our manuscript (PONE-D-25-10509) titled: **“Exosomes combined with extra virgin olive oil reduces lipogenesis, oxidative stress, and inflammation in non-alcoholic fatty liver disease model”**. The whole manuscript has been revised carefully, and we have considered all your comments. These revisions are highlighted in the manuscript by track changes and are summarized below.

**Comment 1**	**Response**
Do not use abbreviations in the keywords section.	We agree with your suggestion and have revised the keywords to avoid the use of abbreviations. The use of full terms in the keywords ensures greater clarity. We changed keywords to **“Liver diseases, Lipid accumulation, Oxidative stress, Lipids peroxidation, Olive oil, Antioxidants, Exosome therapy”**.
**Comment 2**	**Response**
In the introduction, you explained that extra virgin oil has important nutritional effects. Given that impurities in the oil are not removed, please also explain if it has anti-nutritional properties.	Thank you for your thoughtful comment. Extra virgin olive oil (EVOO) is a minimally processed oil obtained through mechanical means, and unlike refined oils, it retains a range of minor compounds such as polyphenols, sterols, and other bioactive molecules. While these compounds contribute to EVOO’s well-documented antioxidant, anti-inflammatory, and cardioprotective properties, we acknowledge the importance of addressing potential anti-nutritional concerns due to its unrefined nature. Upon review of the scientific literature, EVOO is generally not associated with significant anti-nutritional effects. However, in rare cases, certain minor components such as phenolic compounds in excessive concentrations can potentially interfere with nutrient absorption or bioavailability (e.g., by binding to proteins or minerals). These effects are typically not clinically relevant at dietary levels and are outweighed by the health-promoting properties of these same compounds. Additionally, EVOO does not contain harmful processing residues or trans fats found in some refined oils, and any naturally occurring “impurities” are largely considered bioactive constituents rather than contaminants. We added a paragraph in the introduction section of the revised manuscript concerning these points.
**Comment 3**	**Response**
Could the special properties of extra virgin oil be due to compounds other than fatty acids and total antioxidant?	The beneficial properties of extra virgin olive oil (EVOO) cannot be attributed solely to its fatty acid profile and total antioxidant capacity. In addition to its high content of monounsaturated fatty acids particularly oleic acid and antioxidant components like vitamin E, EVOO contains a diverse array of minor bioactive compounds that contribute significantly to its health-promoting properties. These include polyphenols (e.g., hydroxytyrosol, oleuropein, tyrosol): which exhibit strong anti-inflammatory, antimicrobial, and cardioprotective effects. Secoiridoids (e.g., oleocanthal and oleacein): with demonstrated anti-inflammatory and neuroprotective properties, including COX-inhibitory activity similar to that of ibuprofen. Furthermore, the phytosterols and triterpenes, which contribute to

	lipid-lowering and anti-inflammatory actions. These components are unique to EVOO and are largely lost during the refining process of other olive oils. Their synergistic action is believed to enhance the biological effects of EVOO beyond what would be expected from fatty acids or total antioxidant capacity alone.<br>To address this important point, we have included additional detail in the revised introduction highlighting the contribution of these non-lipid minor constituents to the overall bioactivity of EVOO.<br>EVOO is more than just a source of healthy fats. The interaction of fatty acids with a range of minor compounds enhances its therapeutic potential. The inclusion of these compounds distinguishes EVOO from other edible oils and explains many of its superior effects in conditions such as inflammation, oxidative stress, and metabolic disorders, including non-alcoholic fatty liver disease (NAFLD).
**Comment 4**	**Response**
Did you determine the composition of extra virgin oil?	In this study, we did not perform a full compositional analysis of the extra virgin olive oil (EVOO) used. However, the EVOO was sourced from a certified supplier, and we used a product that met the International Olive Council (IOC) standards for extra virgin olive oil. This includes specific criteria for free acidity, peroxide value, UV absorbance, and sensory characteristics, ensuring its quality and authenticity. While we acknowledge that the exact composition of EVOO can vary depending on factors such as olive variety, geographic origin, and processing methods, we relied on a commercially available, well-characterized EVOO known for its typical composition—including high levels of oleic acid, polyphenols, tocopherols, and other minor bioactive compounds. To improve the rigor of future studies, we agree that a detailed compositional analysis (e.g., GC-MS, HPLC) would be beneficial to correlate specific bioactive components with observed biological effects. We have now included a statement in the Limitations section to acknowledge this point.
**Comment 5**	**Response**
It is not necessary to use "showing" at the beginning of the names of arguments and forms.	We appreciate your suggestion and understand that the use of “showing” at the beginning of argument or figure descriptions can be redundant or overly descriptive. Based on your feedback, we have revised the manuscript to remove unnecessary uses of “showing” in figure captions, table titles, and related text.

We would like to thank you for helping to increase the quality of this paper. We hope the changes that have been made were appropriate and the manuscript can now be accepted for publication. Please address all correspondence to the author indicated below.

Sincerely Yours,

Corresponding author

Prof. Dr. Bassam Alowaish

Dear Reviewer, 2,

Thank you for the review of our manuscript (PONE-D-25-10509) titled: **"Exosomes combined with extra virgin olive oil reduces lipogenesis, oxidative stress, and inflammation in non-alcoholic fatty liver disease model"**. The whole manuscript has been revised carefully, and we have considered all your comments. These revisions are highlighted in the manuscript by track changes and are summarized below.

**Major issues:**	
**Comment 1**	**Response**
Statistical analysis: in the figure legend, "Bars with different letters show statistical significance between groups (P<0.05) using Tukey's HSD." What do different letters exactly mean? a, b, c? Please provide all the p values.	In our figures, different lowercase letters (e.g., a, b, c) above the bars indicate statistically significant differences between groups as determined by Tukey's Honest Significant Difference (HSD) post hoc test, following one-way ANOVA.<br><br>Bars labeled with the same letter (e.g., both labeled "a") indicate no significant difference between those groups. Bars labeled with different letters (e.g., "a" vs. "b") indicate a significant difference (P<0.05). This lettering method is a standard approach in scientific figures to show group-wise comparisons visually and clearly when multiple groups are analyzed.<br><br>We clarify these points in the figure legends and table footnotes as th follows: "Bars with different lowercase letters (e.g., a, b, c) indicate statistically significant differences between groups, as determined by Tukey's HSD post hoc test following one-way ANOVA. Groups sharing the same letter are not significantly different". All (P- values) were provided.
**Comment 2**	**Response**
Related to issue 1. From the bar charts shown in certain figures, the efficacy advantage of combined administration seems to be not significant when compared with olive oil or exosomes.	Thank you for this comment, as highlighted in the manuscript, any between group comparisons were done using a rigorous post-hoc test (Tukey's Honestly Significant Difference), therefore any difference although to the naked eye appears not significant, however statistically the test shows its true significance level. In some cases, the test failed to show significance between groups such as triglycerides between treated groups (any treatment) and control group (shown in Fig.1-A), which is a good indication in this case that any used treatment can reduce TGs levels to approach near normal levels. Same observation can be done for Liver MDA concentration in Fig.2-B, where all treated groups showed no significant difference from the control group, which also means that there is no difference in the lipid peroxidation between control and treated groups, which is also a good indication that our different treatments were able, to a good extent, to protect liver cells from the oxidative damage.

Comment 3	Response
In the experimental design, the hyperlipidemia group was treated with 50μg of exosomes derived from BM-MSCs once a week by intraperitoneal injection. Why did the author use i.p., instead of i.v.? In addition, how to determine the dosage of exosomes?	We chose an intraperitoneal (i.p.) injection for the following reasons: technical feasibility and safety: i.p. injection is technically easier and safer to perform repeatedly in small animals (such as mice or rats) compared to i.v. injection, which can be more invasive and stressful for the animals. Several studies have demonstrated that i.p. injection allows for effective systemic absorption and biodistribution of exosomes, including delivery to liver tissue, which is a primary target in non-alcoholic fatty liver disease (NAFLD) models. Moreover, previous preclinical studies using mesenchymal stem cell (MSC)-derived exosomes for metabolic or liver diseases have used i.p. administration with effective outcomes (e.g., references can be added to support this). Therefore, the i.p. route was selected as a practical and effective method for systemic delivery of exosomes in our NAFLD model.<br>The exosome dosage of 50 μg/week was based on prior studies, we reviewed several published animal studies involving BM-MSC-derived exosomes for liver and metabolic disorders, which typically used dosages ranging from 10–100 μg per injection, depending on the disease model, treatment duration, and route of administration. In preliminary trials (or pilot data, if available), we observed that 50 μg/week was well-tolerated and resulted in measurable therapeutic effects without signs of toxicity or adverse reactions. Furthermore, the dose was quantified based on total protein content, which is a standard method for approximating exosome amounts in the absence of universally accepted dosage standards. This approach is consistent with the methods reported in other exosome-related studies.<br>We acknowledge that exosome dosing remains an evolving field without standardized guidelines. We have included a statement in the revised manuscript to suggest that further dose-response studies would be beneficial in future research.
**Comment 4**	**Response**

The phenotype of NAFLD in the rat model is not obvious. Is there any index of NAFLD in rats fed with high fat diet? Or is the disease model just hyperlipidemia?	Thank you for this critical observation and opportunity to clarify the disease model used in our study. We agree that it is essential to distinguish between hyperlipidemia and a full NAFLD phenotype, especially in preclinical models. While hyperlipidemia is often an early metabolic change induced by a high-fat diet (HFD), non-alcoholic fatty liver disease (NAFLD) requires additional evidence of hepatic steatosis, inflammation, and possibly early fibrosis to be clearly defined.<br>In our study, rats were fed a high-fat diet (HFD) for 8 weeks, which is a widely accepted method for inducing NAFLD-like features. We evaluated liver histology, which showed macrovesicular steatosis, hepatocyte ballooning, and in some cases mild inflammatory infiltration, all consistent with NAFLD criteria. We used assessments to confirm the NAFLD phenotype including Serum biochemical markers such as ALT, AST, total cholesterol, triglycerides, and LDL-C to assess liver injury and systemic lipid metabolism. Histopathological evaluation to confirm lipid accumulation and liver tissue changes.
**Minor issues:**	
**Comment 1**	**Response**
Figure 4: labels arrow, arrowhead correctly.	We have carefully reviewed Figure 4 and updated the figure legend and image annotations to ensure that arrows and arrowheads are clearly labeled and accurately correspond to the structures or features they are intended to indicate
**Comment 2**	**Response**
Make sure the references are appropriate.	We have carefully reviewed all the references cited in the manuscript to ensure their scientifically relevant and up to date
**Comment 3**	**Response**
Some sentences are repetitive, such as page 15, line 8-10.	We have carefully reviewed page 15, lines 8–10, as well as the surrounding text, and agree that the sentences in question were repetitive in content and phrasing. We have revised this section to remove redundancy and improve clarity, ensuring that each sentence contributes new or necessary information without restating previous points.

We would like to thank you for helping to increase the quality of this paper. We hope the changes that have been made were appropriate and the manuscript can now be accepted for publication. Please address all correspondence to the author indicated below.

Sincerely Yours,

Corresponding author

Prof. Dr. Bassam Alowaish
