# Supplementary material for: Exosomes combined with extra virgin olive oil reduces lipogenesis, oxidative stress, and inflammation in non-alcoholic fatty liver disease model
Source: PLoS One. 2025 Oct 27;20(10):e0333698. doi: 10.1371/journal.pone.0333698 (PMC12558531; doi:10.1371/journal.pone.0333698)
Supplement: Attachment — Submitted filename: Comments.docx [file pone.0333698.s001.docx]

This manuscript is " Exosomes combined with extra virgin olive oil reduces lipogenesis, oxidative stress, and inflammation in non-alcoholic fatty liver disease model ". The topic of this manuscript is interesting and suitable for publication in the journal. However, some points need explanation, which are mentioned below:

1. Do not use abbreviations in the keywords section.
2. In the introduction, you explained that extra virgin oil has important nutritional effects. Given that impurities in the oil are not removed, please also explain if it has anti-nutritional properties.
3. Could the special properties of extra virgin oil be due to compounds other than fatty acids and total antioxidant?
4. Did you determine the composition of extra virgin oil؟
5. It is not necessary to use "showing" at the beginning of the names of arguments and forms.
