# Supplementary material for: Exosomes combined with extra virgin olive oil reduces lipogenesis, oxidative stress, and inflammation in non-alcoholic fatty liver disease model
Source: PLoS One. 2025 Oct 27;20(10):e0333698. doi: 10.1371/journal.pone.0333698 (PMC12558531; doi:10.1371/journal.pone.0333698)
Supplement: Attachment — Submitted filename: Response to Reviewer.docx [file pone.0333698.s003.docx]

**Dear Reviewer 2,**

Thank you for the review of our manuscript (PONE-D-25-10509) titled: **“Exosomes combined with extra virgin olive oil reduces lipogenesis, oxidative stress, and inflammation in non-alcoholic fatty liver disease model”**. The whole manuscript has been revised carefully, and we have considered all your comments. These revisions are highlighted in the manuscript by track changes and are summarized below.

| **Comment 1** | **Response** |
| --- | --- |
| Related to comment 3:  About the injection method and dosage of exosomes, please add references to support this and discuss. | We thank the reviewer for this important point. We have added clarifying text to the Methods and Discussion explaining our choice of route and dose and have cited representative preclinical studies and reviews. Briefly, the chosen dose of **50 µg total exosomal protein per rat/week (i.p.)** is consistent with multiple published rat studies that reported therapeutic effects at similar protein-based doses (dose ranges reported in the literature vary with model and route, e.g. ~50–200 µg per rat in some dose–response studies). The intraperitoneal route is commonly used in small animal exosome work for its practicality and reproducible systemic exposure (via lymphatic absorption). We added citations to dose-response and biodistribution literature to support these points |

We would like to thank you for helping to increase the quality of this paper. We hope the changes that have been made were appropriate and the manuscript can now be accepted for publication. Please address all correspondence to the author indicated below.

**Sincerely Yours,**

**Corresponding author**

Prof. Dr. Bassam Alowaish
